# ^13^C_carbene_ nuclear magnetic resonance chemical shift analysis confirms Ce^IV^=C double bonding in cerium(iv)–diphosphonioalkylidene complexes†

**DOI:** 10.1039/d3sc04449a

**Published:** 2023-12-06

**Authors:** Cameron F. Baker, John A. Seed, Ralph W. Adams, Daniel Lee, Stephen T. Liddle

**Affiliations:** a Department of Chemistry, The University of Manchester Oxford Road Manchester M13 9PL UK daniel.lee@manchester.ac.uk steve.liddle@manchester.ac.uk; b Department of Chemical Engineering, The University of Manchester Oxford Road Manchester M13 9PL UK

## Abstract

Diphosphonioalkylidene dianions have emerged as highly effective ligands for lanthanide and actinide ions, and the resulting formal metal–carbon double bonds have challenged and developed conventional thinking about f-element bond multiplicity and covalency. However, f-element–diphosphonioalkylidene complexes can be represented by several resonance forms that render their metal–carbon double bond status unclear. Here, we report an experimentally-validated ^13^C Nuclear Magnetic Resonance computational assessment of two cerium(iv)–diphosphonioalkylidene complexes, [Ce(BIPM^TMS^)(ODipp)_2_] (1, BIPM^TMS^ = {C(PPh_2_NSiMe_3_)_2_}^2−^; Dipp = 2,6-diisopropylphenyl) and [Ce(BIPM^TMS^)_2_] (2). Decomposing the experimental alkylidene chemical shifts into their corresponding calculated shielding (*σ*) tensor components verifies that these complexes exhibit Ce=C double bonds. Strong magnetic coupling of Ce=C σ/π* and π/σ* orbitals produces strongly deshielded *σ*_11_ values, a characteristic hallmark of alkylidenes, and the largest ^13^C chemical shift tensor spans of any alkylidene complex to date (1, 801 ppm; 2, 810 ppm). In contrast, the phosphonium-substituent shielding contributions are much smaller than the Ce=C σ- and π-bond components. This study confirms significant Ce 4f-orbital contributions to the Ce=C bonding, provides further support for a previously proposed inverse-trans-influence in 2, and reveals variance in the 4f spin–orbit contributions that relate to the alkylidene hybridisation. This work thus confirms the metal–carbon double bond credentials of f-element–diphosphonioalkylidenes, providing quantified benchmarks for understanding diphosphonioalkylidene bonding generally.

## Abstract 2

A combined solid-state NMR and DFT computational shielding study confirms the presence of Ce=C double bonds in cerium–diphosphonioalkylidene complexes.

## Graphical abstract

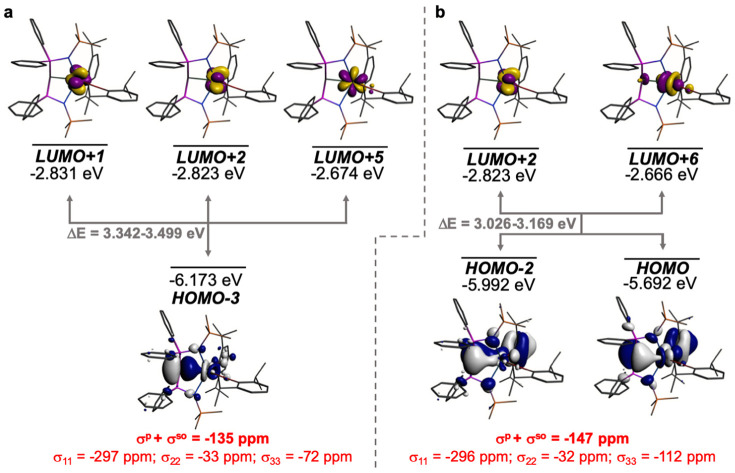

## Introduction

**Fig. 1:**
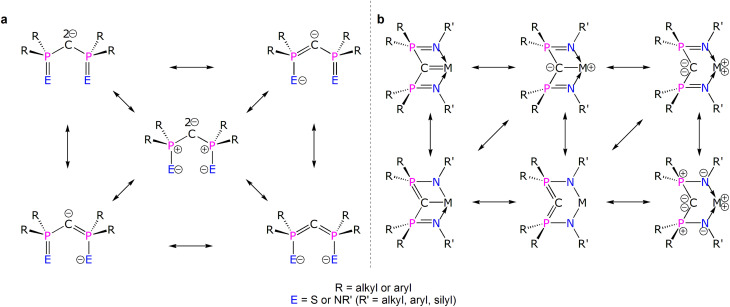
Various electronic resonance forms of {(R_2_PE)_2_C^2−^} and {(R_2_PNR′)_2_CM} structures. (a) Principal resonance forms for {(R_2_PE)_2_C^2−^}. (b) Selected electronic structure representations of a {(R_2_PNR′)_2_CM} unit. The two-headed arrows emphasise the electronic structure inter-relationships but are not exhaustively representative.

Diphosphonioalkylidene (methanediide) ligands, {(R_2_PE)_2_C^2−^} (R = alkyl or aryl; E = S or NR’; R' = silyl, aryl, alkyl), have proven to be popular carbene ligands for metals across the Periodic Table, and in particular they have been effective in developing formal M=C (M = lanthanide and actinide) double bond interactions that have challenged and developed conventional thinking on f-element multiple bonding and covalency.^1–10^ The E = NR′ variant, the Bis(IminoPhosphorano)Methanediide (BIPM) class, has proven to be very versatile, supporting formal M=C double bonds over M oxidation states +3 to +6, novel bonding motifs, reactivity, and magnetism, and even transuranium derivatives.^11–25^ However, the polarised nature of electropositive metal bonding and the various resonance forms that can be drawn for these methanediides (Fig. 1a)^4^ raises fundamental questions over how best to pictorially represent BIPM bonding to metals and then what those representations actually mean (Fig. 1b).^26^ This is because any M=CR_2_ linkage features a C-atom that is supported to various extents by tensioned M and R stabilising contributions; the phosphonium-substituents can in principle stabilise the C-centres of M=C_BIPM_ complexes, that is take on ylide P=C double bond character, thus diminishing the extent of M=C double bond character. Consequently, the extent of M=C double bond character of diphosphonioalkylidene complexes has remained open to qualitative interpretation.^27–30^ Thus, quantification to provide a more rigorous descriptive framework is required.

In recent years, ^13^C NMR spectroscopic studies of transition metal alkylidenes have delivered a comprehensive understanding of M=CR_2_ double bonds.^31–35^ In particular, the isotropic chemical shift (*δ*_iso_) is intimately dependent on the shielding (*σ*) tensors, and a signature feature of alkylidene complexes which has emerged is that the *σ*_11_ tensor component, which is in the M=CR_2_ plane and orthogonal to the M=C σ- and π-bond principal axes, is substantially deshielded due to strong magnetic coupling of the M=C σ/π* and π/σ* orbitals.^31^ When considering applying that approach to diamagnetic lanthanide- and actinide-BIPM complexes, where data are available they exhibit a wide range of ^13^C NMR C_carbene_ chemical shifts^4,7,8,10^ implying a varied range of bonding scenarios where the more deshielded the C_carbene_*δ*_iso_ value is the more multiple bond character it will likely have to the metal, if not disproportionately shifted by spin orbit effects. However, in contrast to transition metal alkylidenes^31–36^ a detailed dissection of the shielding tensors beyond calculated *δ*_iso_ values has remained largely untested for f-element–BIPM complexes.^30,37,38^

**Fig. 2:**
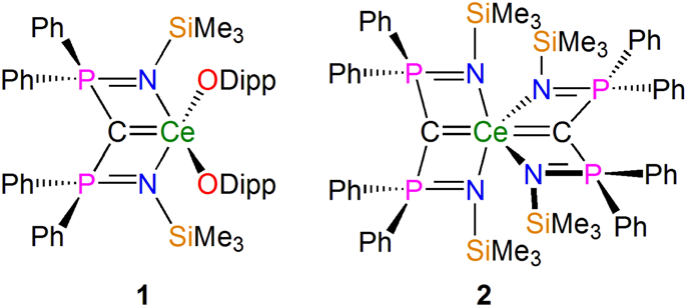
Cerium-carbene complexes 1 and 2. These complexes are the two subject molecules of this study. Dipp = diisopropylphenyl.

In recent years NMR spectroscopy has emerged as a powerful tool for quantifying f-element chemical bonding when the individual contributions to the shielding tensors are analysed in detail,^39^ because the chemical shifts of a wide range of nuclei have proven to be very sensitive to the nature of their interactions with f-block ions.^30,37,38,40–62^ Recently some of us,^56,58,62^ and others,^53,54^ demonstrated that ^15^N, ^29^Si, and ^31^P NMR spectroscopies combined with computational analysis of chemical shielding tensor properties provides powerful probes of f-element–ligand covalency, so our attention turned to examining BIPM–f-element complexes using ^13^C NMR spectroscopy. We focus on two cerium(iv)-carbene complexes [Ce(BIPM^TMS^)(ODipp)_2_] (1, BIPM^TMS^ = {C(PPh_2_NSiMe_3_)_2_}^2−^; Dipp = 2,6-diisopropylphenyl)^15,18^ and [Ce(BIPM^TMS^)_2_] (2) (Fig. 2).^19^ Using ^13^C–^31^P 2D solution NMR spectroscopy the ^13^C *δ*_iso_ of the carbene centres in 1 and 2 were previously determined to be 324.6 (*J*_PC_ = 149 Hz) and 343.5 (*J*_PC_ = 170 Hz) ppm, respectively. These downfield *δ*_iso_ values are unusually highly deshielded and well into the usual range (200–400 ppm) of alkylidenes,^36^ and a range of spectroscopic, magnetic, and computational methods consistently describe 1 and 2 as having no appreciable temperature independent paramagnetism (TIP) and being closed-shell singlet (*i.e.* not multi-reference) formulations.^18,19^ These two complexes therefore represent ideal benchmarks from which to quantify the nature of the Ce=C_BIPM_ bonds and so inform the debate that surrounds the multiple bonding aspect of BIPM complexes generally.

Here, we report an assessment of the shielding tensors that underpin the *δ*_iso_ values for 1 and 2. This work confirms that Ce=C double bond interactions are indeed present in 1 and 2, revealing dominant *σ*_11_ data that are the hallmark of alkylidenes, and the largest tensor spans of any metal-alkylidene complex. The data quantifies the relative extent of Ce=C σ- and π-bond stabilisation with respect to the smaller phosphonium-substituent contributions, provides further support for the previously proposed inverse-*trans*-influence (ITI) in 2, and reveals variance in the spin–orbit-induced spin-polarisation of the C_carbene_ that can be related to the σ- and π-components and their variable levels of 2s *vs.* 2p character. Overall, this work confirms the M=C double bond credentials of these diphosphonioalkylidene complexes and provides quantified benchmarks for diphosphonioalkylidene bonding more generally.

## Results

### Solid-state NMR spectroscopy of 1 and 2

**Fig. 3:**
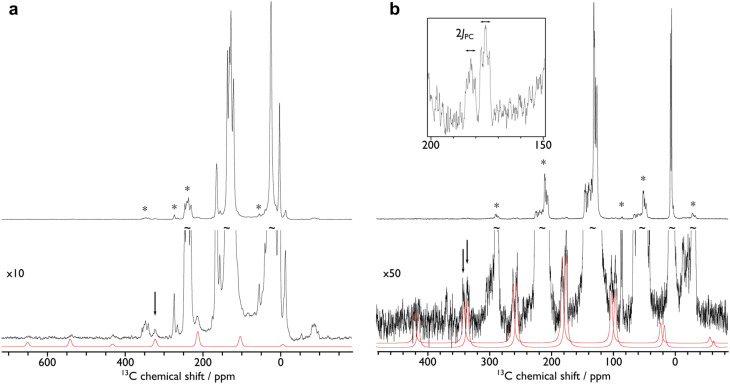
{^1^H-}^13^C CPMAS NMR spectra of (a) 1 and (b) 2. The fits of the carbene chemical shift anisotropy parameters are given in red, black arrows highlight the isotropic chemical shifts, and asterisks (*) denote spinning sidebands of non-carbene signals. The inset of (b) shows an expansion of carbene spinning sidebands for 2 where the ^31^P–^13^C *J*-coupling can be measured (170 ± 20 Hz). The spectra were recorded at ambient temperature at 9.4 T, using MAS frequencies of 11 kHz (1) and 8 kHz (2).

In order to confirm the solution *δ*_iso_ values for 1 and 2 and provide experimental benchmarking for the computational analysis of 1 and 2, solid-state {^1^H-}^13^C CPMAS NMR spectra were recorded permitting fitting of the chemical shift anisotropy (CSA) parameters (Fig. 3). The solid-state ^13^C *δ*_iso_ of the carbene centre in 1 is 322.5 ppm, and for 2 two values of 334.5 and 341.5 ppm were determined, consistent with the two different Ce=C distances found in the solid-state structure of 2 (Ce=C = 2.385(2) and 2.399(3) Å);^19^ both sets of solid-state *δ*_iso_ values are in excellent agreement with the solution *δ*_iso_ values (*vide supra*). Moreover, the solid-state *J*_PC_ of 2 (170 ± 20 Hz) could be extracted, owing to the high crystallinity and corresponding small ^13^C linewidths of the sample, and is consistent with the solution *J*_PC_ value (*vide supra*). Fitting of the CSA parameters (Fig. 3) produced *δ*_11_, *δ*_22_, *δ*_33_, span (*Ω*), and skew (*κ*) values of 815.9, 136.7, 14.9, 801 ppm, and −0.70 for 1 and 816.5/823.5, 180.6/187.6, 6.4/13.4, 810/810 ppm, and −0.57/−0.57 for the two carbene centres in 2. For 1 and 2 the spans of the chemical shift tensor *Ω* values of approximately 801 and 810/810 ppm are the largest to date for any metal-alkylidene.^31–35^ The *κ* values are consistent with the presence of M=C double bonds, with 2 being close to the ideal of 0.5 and the deviation for 1 being accounted for by the slight pyramidalisation of the carbene in that complex. The extremely large deshielding of *δ*_11_ for these M=C carbenes suggests strong magnetic coupling between occupied and vacant orbitals, in particular 
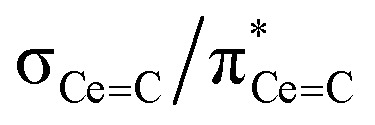
 and 
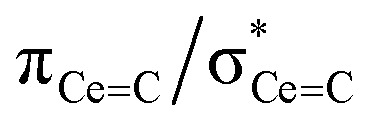
.

### Computational benchmarking of the ^13^C_carbene_ NMR spectroscopic properties of 1 and 2

The computational assessment began by taking the solid-state crystallographic coordinates of 1 and 2 and geometry optimising the H-atom positions whilst constraining the heavy atom positions at the BP86 TZ2P all-electron ZORA relativistic level (Tables S1 and S2†). Using the resultant atom coordinates scalar relativistic (SR) and spin–orbit relativistic (SOR) single point energy calculations for 1 and 2 were conducted using a range of functionals (BP86, PBE0-HF25 (default HF setting in ADF), PBE-HF40, B3LYP-HF20 (default HF setting in ADF), B3LYP-HF30, B3LYP-HF35, and B3LYP-HF40) and in turn those data were used to compute the SR and SOR ^13^C_carbene_*δ*_iso_ values in a benzene solvent continuum (Tables S3 and S4†). For 1 the best agreement was found using the B3LYP-HF20 functional, where the computed SR ^13^C_carbene_*δ*_iso_ value of 298.4 ppm shifts to 324.9 ppm in the SOR calculation. Both ^13^C_carbene_*δ*_iso_ values for 2 were computed, but since the variance for pairs of values was ≤0.4 ppm we present average data; the B3LYP-HF30 functional gave the best agreement, with the computed SR ^13^C_carbene_*δ*_iso_ value of 258.9 ppm shifting to 341.8 ppm in the SOR calculation. These values are in excellent agreement with the solution and solid-state *δ*_iso_ data for 1 and 2, and the use of slightly different functionals (10% difference in HF mixing) was considered acceptable.^63^ From these calculations we extracted the *δ*_11_, *δ*_22_, *δ*_33_, *Ω*, and *κ* values for 1 and 2; the calculated values are 834.2, 132.5, 7.9, 842.1 ppm and −0.69 for 1 and 836.6, 190.7, −1.9, 838.5, and −0.54 (av.) for 2, which fit well with the experimental MAS NMR data.

### Molecular orbital and natural bond orbital benchmarking of 1 and 2

Having established that the B3LYP-HF20 and -HF30 functionals satisfactorily reproduce the experimental ^13^C_carbene_*δ*_iso_ and CSA values of 1 and 2, respectively, their electronic structures were reanalysed at those levels of theory. In both cases the Molecular Orbital (MO) manifolds for 1 and 2 are qualitatively quite similar to the previously published BP86 data,^15,18,19^ revealing Ce=C σ- and π-bonding combinations. The Nalewajski-Mrozek Ce=C bond orders for 1 and 2 are computed to be 1.10 and 1.16, respectively, consistent with a two-fold bonding interaction where each component has a sub-one bond order, *i.e.* each component is polarised. These B3LYP values are very similar to the BP86 values of 1.10 for both 1 and 2. However, there is much greater mixing of orbital coefficients across the B3LYP frontier MOs of 1 and 2 compared to the corresponding BP86 calculations, a situation also encountered when comparing BP86 and B3LYP data in the aforementioned terminal uranium(vi)-nitride ^15^N NMR spectroscopic study.^58^

**Fig. 4:**
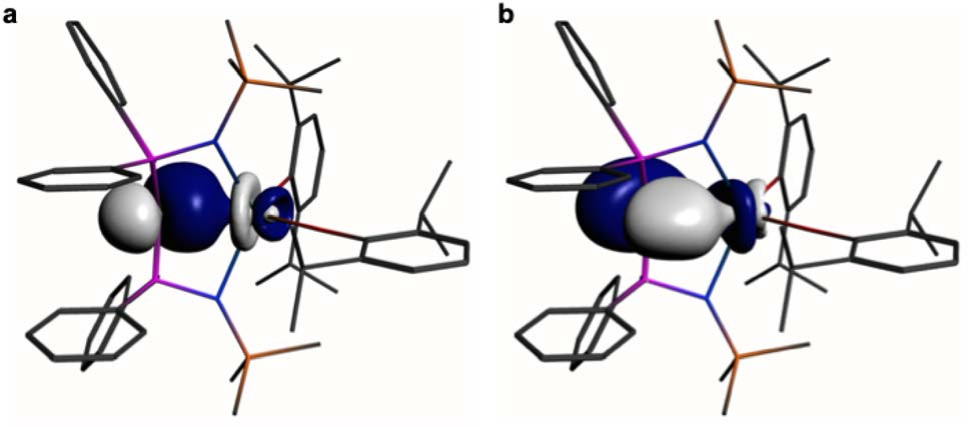
Natural Bond Orbitals (NBOs) of 1 at the B3LYPHF20 level. (a) The Ce=C σ-NBO. (b) The Ce=C π-NBO. Hydrogen atoms are omitted for clarity.

**Fig. 5:**
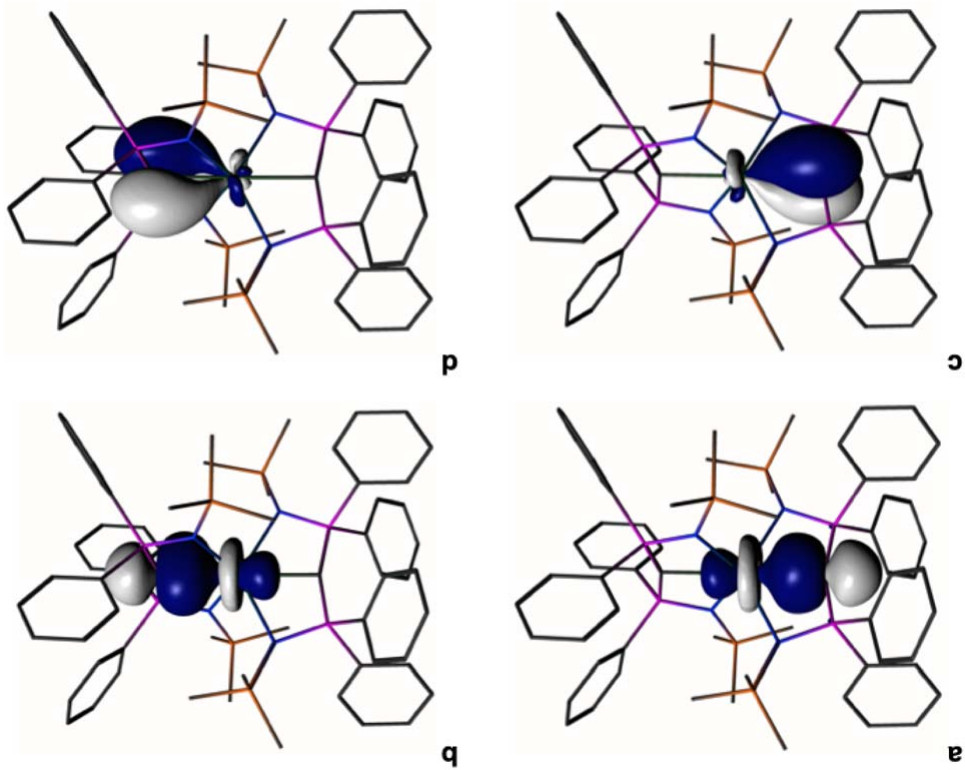
Natural Bond Orbitals (NBOs) of 2 at the B3LYPHF30 level. (a and b) The two Ce=C σ-NBOs. (c and d) The two Ce=C π-NBOs. Hydrogen atoms are omitted for clarity.

In order to provide a chemically localised and hence more instructive model than the delocalised MO description, the Natural Bond Orbitals (NBOs) of 1 and 2 were examined using the B3LYP-HF20 and -HF30 functionals, respectively. In both cases NBO identifies clear-cut σ- and π-bonding interactions constituting Ce=C double bonding interactions (Fig. 4 and 5). For 1, the Ce=C σ-bond is found to be 13% Ce (6s/6p/5d/4f: 1/0/32/67%) and 87% C_carbene_ (2s/2p: 15/85%) character. The Ce=C π-bond in 1 is similarly polarised being composed of 11% Ce (6s/6p/5d/4f: 1/1/31/67%) and 89% C_carbene_ (2s/2p: 2/98%). For 2, the Ce=C σ-bond is 15% Ce (6s/6p/5d/4f: 3/0/47/50%) and 85% C_carbene_ (2s/2p: 11/89%) but the π-bonds are more polarised at 7% Ce (6s/6p/5fd/4f: 0/0/53/47%) and 93% C_carbene_ (2s/2p: 0/100%) character. We note in passing that these NBO data are in good agreement with the previously reported BP86-NBO data (see Tables S5 and S6† for BP86–B3LYP comparisons).^15,18,19^ Given that these calculations satisfactorily reproduce the experimentally determined *δ*_iso_ spectroscopic data, they: (i) quantify significant contributions of 5d- and 4f-orbital bonding character for Ce; (ii) acknowledging that the bonding is polarised, support the Ce=C double bonding interaction description.

### Computational chemical shielding analysis of 1 and 2

In order to contextualise the following discussion, it is useful to relate the *δ*_iso_ to its isotropic shielding (*σ*_iso_) and in turn *σ*_iso_ to its constituent diamagnetic (*σ*^d^), paramagnetic (*σ*^p^) and spin–orbit (*σ*^so^) shielding components. The *δ*_iso_ is derived from the *σ*_iso_ of the NMR nucleus being considered when adjusted for the *σ*_iso_ and *δ*_iso_ of the NMR nucleus standard reference (here the ^13^C nucleus of CH_4_ at *δ*_iso_ −4.3 ppm).

Ramsey's formalism, eqn (1), relates NMR interactions to a quantum-mechanical perspective by decomposing magnetic shielding contributions into *σ*^d^ and *σ*^p^ components. These parameters are dependent on electron orbital angular momenta,^64–66^ and whilst this does not directly translate to the MO approach of hybrid DFT (B3LYP), it provides a framework with which to rationalise NMR magnetic shielding calculations when *σ*^so^ contributions are included,^67^eqn (2).

1 *σ*_iso_ = *σ*^d^ + *σ*^p^

2 *σ*_iso_ = *σ*^d^ + *σ*^p^ + *σ*^so^

The ^13^C_carbene_*σ*^d^ values for 1 and 2 are 268.6 and 258.9 ppm, respectively. As expected, there is relatively little variation for these values, since *σ*^d^ derives principally from tightly-bound core electron densities that respond little to valence-level perturbations.^67^ The ∼10 ppm variance within the ^13^C_carbene_*δ*_iso_ values of >320 ppm can be considered to be minor and thus negligible to the discussion.

Turning to the ^13^C_carbene_*σ*^so^ data, the values for 1 and 2 are −23.6 and −79.7 ppm, respectively, which is consistent with *σ*^so^ contributions in other Ce^IV^–C complexes,^55^ but in passing a *σ*^so^ of close to −80 ppm is indicative of significant 4f-orbital character in the bonding, which is notable given the usual ‘core-like’ description of 4f-orbitals. This likely reflects the strong Ce=C σ-bonding in 2, see below. These are clearly not insignificant contributions to the ^13^C_carbene_*σ*_iso_ values of 1 and 2, but given the observed ^13^C_carbene_*δ*_iso_ values of >320 ppm, which would be >280 ppm in the absence of spin–orbit effects it is clear that whilst the *σ*^so^ data should not be ignored they can be considered to be secondary to the primary determinant of the ^13^C_carbene_*δ*_iso_ values, which is the *σ*^p^ values.

The *σ*^p^ term can be presented in reduced form as:3
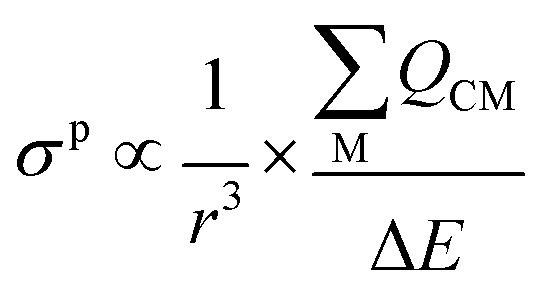
In eqn (3)*r* is the radial expansion of the shielding electrons from the nucleus being examined, C denotes the NMR nucleus (the C_carbene_), 
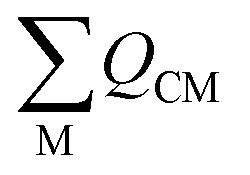
 is the sum of the charge density and bond order matrix elements over the relevant atoms (M), and Δ*E* is the energy separation between the ground and excited states in question.^39,46,64–66^

The *σ*^p^ term is inversely proportional to the energy gap between the occupied and virtual orbitals that become magnetically coupled in the presence of an externally applied magnetic field, so smaller Δ*E* gaps produce larger *σ*^p^ values. However, examination of the HOMO–LUMO gaps of 1 and 2 shows that they are unremarkable (2.846–4.218 eV in B3LYP calculations) and so a disproportionate effect on *σ*^p^ from Δ*E* can be discounted.

Field-induced magnetic mixing of the ground state with low-lying, thermally inaccessible, paramagnetic states in 1 and 2, that is TIP, has been previously found to be negligible^18,19^ and multi-reference calculations on 1 and 2 showed little multi-reference character.^18,19^ This suggests that any TIP effects on the *σ*^p^ term will be modest,^52,58^ and thus the 
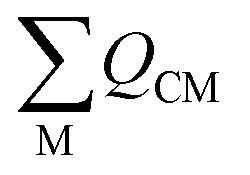
 term will, like the Δ*E* term, not introduce a disproportionate effect on *σ*^p^.

Turning to the remaining *r*^3^ term, *σ*^p^ is inversely proportional to *r*^3^. This is because as a nucleus (M) withdraws charge from the NMR nucleus (C) the C valence orbitals contract due to the increased electron deficiency at C. Thus, the 1/*r*^3^ term becomes larger (*i.e.* the NMR nucleus is more deshielded) resulting in a larger *σ*^p^ term. Put another way, the larger the bond order of, so more covalent, the bond involving the NMR nucleus the larger *σ*^p^ becomes.^46^ In this context, recalling the *δ*_iso_ values of 1 and 2, the *σ*^p^ values of −382.1 and −333.1 ppm are large, and hence significant, and consistent with the presence of Ce=C double bond interactions.

### Molecular orbital shielding analysis

The external field-induced magnetic coupling of occupied and virtual orbitals must be symmetry-allowed, since the angular momentum operators belong to the same irreducible representations as the rotational operators. The contributions to deshielding can be distributed over many components because the MOs are often delocalised, and so are difficult to fully identify. Furthermore, as mentioned above, the B3LYP MO manifold contains ‘split’ Ce=C σ- and π-bonding combinations across different MOs. However, noting the above framework and using the ADF NMR analysis package enables identification and recombination of the principal components that contribute to the *σ*^p^ term of 1 and 2.

**Fig. 6:**
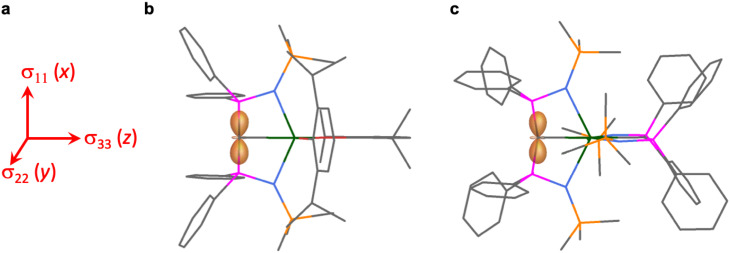
Plots of the *σ*_11_, *σ*_22_, and *σ*_33_ tensor components for 1 and 2 as a shielding surface. (a) The orientation of the tensor components *σ*_11_, *σ*_22_, and *σ*_33_ which approximately align along the principal axes *x*, *y*, and *z*. (b) Shielding surface for the carbene of 1. (c) Shielding surface for one of the carbenes of 2 (the other is essentially the same). The shielding surfaces are represented using the ovaloid convention where the distance from the C atom to a point on the surface is proportional to the chemical shift when the magnetic field is aligned along that direction in space. The shading of the surface denotes the sign of the shift where light orange is positive and orange is negative. Key: green = Ce; magenta = P; orange = Si; red = O; blue = N; grey = C. Hydrogen atoms are omitted for clarity.

**Fig. 7:**
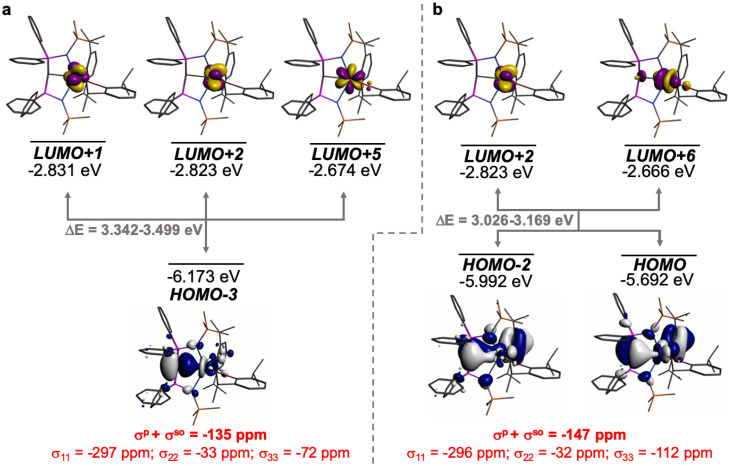
Dominant occupied and virtual molecular orbitals that contribute to the *σ*_iso_ and hence *δ*_iso_ values of 1 by magnetic field-induced magnetic coupling. (a) Magnetic coupling of the occupied Ce=C σ-bond with unoccupied Ce=C π*- and 4f-orbitals. (b) Magnetic coupling of the occupied Ce=C π-bond with unoccupied Ce=C σ*- and f-orbitals. Hydrogen atoms are omitted for clarity. Note, the Ce=C π-bond is split into two molecular orbital representations due to mixing with different aryloxide and BIPM^TMS^ N-lone pair orbital coefficients. The isotropic shielding values for the individual bonding components are given in red, and each is broken down into its constituent *σ*_11_, *σ*_22_, and *σ*_33_ principal component contributions.

**Fig. 8:**
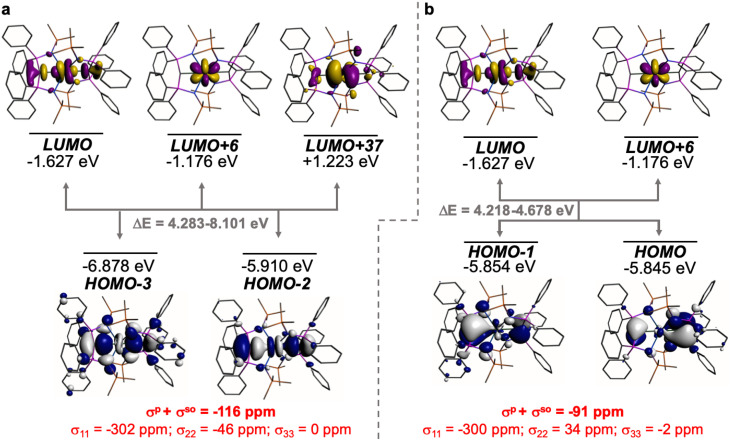
Dominant occupied and virtual molecular orbitals that contribute to the *σ*_iso_ and hence *δ*_iso_ values of 2 by magnetic field-induced magnetic coupling. (a) Magnetic coupling of the occupied Ce=C σ-bonds with unoccupied Ce=C π*- and 4f-orbitals. (b) Magnetic coupling of the occupied Ce=C π-bonds with Ce=C σ*- and f-orbitals. Hydrogen atoms are omitted for clarity. Note, in each case the individual Ce=C σ- and π-bonding interactions are delocalised across two molecular orbitals each due to their symmetric and asymmetric symmetry adapted linear combinations. The isotropic shielding values for the individual bonding components are given in red, and each is broken down into its constituent *σ*_11_, *σ*_22_, and *σ*_33_ principal component contributions.

The shielding effects can be understood in terms of the rotated orbital model,^31,68–71^ which considers the action of the angular momentum operator on magnetically coupled occupied and virtual orbitals, which can be visualised as a 90° rotation of an idealised occupied C p-orbital to mix with an orthogonal vacant orbital. This has been comprehensively described elsewhere for alkylidenes,^31^ but of pertinence to the results here in brief the computed orientations of the ^13^C *σ*_11_, *σ*_22_, and *σ*_33_ shielding tensor principal components of 1 and 2 are shown in Fig. 6, where it can be seen that they align closely to the principal axes (*x*, *y*, and *z*). Thus, magnetic coupling of the σ and π* and π and σ* orbitals will correspond to rotation about the *x* axis resulting in *σ*_11_ deshielding along the *x* axis. The results of the MO analysis are presented in Fig. 7 and 8. For 1 and 2, for a given BIPM^TMS^ ligand the occupied Ce=C σ-bond mixes with unoccupied Ce=C π*- and 4f-orbitals and the Ce=C π-bond mixes with unoccupied Ce=C σ*- and f-orbitals. In all cases, the dominant individual σ contribution to the *σ*_iso_ is *σ*_11_, which is deshielded due to strong σ/π* and π/σ* magnetic couplings that are orthogonal to the *σ*_11_ direction (*x* axis), and this is a signature feature of alkylidene complexes. Thus, for both 1 and 2 the principal magnetic coupling of orbitals that is responsible for the shielding tensors at the carbene centres derives from orbitals associated with the Ce=C linkage as found analogously in transition metal alkylidenes.^31–35^

### Natural localised molecular orbital shielding analysis

Whilst the MO analysis above provides a qualitatively instructive framework to probe the field-induced magnetic coupling of occupied and virtual orbitals, the analysis is incomplete due to the delocalised nature of MOs. In order to derive a more complete picture the shielding data in terms of the Natural Localised Molecular Orbitals (NLMOs) were analysed,^72,73^Table 1. The NLMOs for 1 and 2 are in each case very similar to the respective NBO data (see Tables S7 and S8† for NBO-NLMO comparisons). Compared to B3LYP, the BP86 data tend to return larger 4f-orbital contributions at the expense of 5d-orbital contributions for Ce. However, the C 2s/2p% and total M/C% values vary little, so since NLMOs are effectively NBOs that have been allowed to expand to include other small orbital coefficients to ensure the orbital occupancy is 100%, the B3LYP NLMO analysis is directly representative of the B3LYP NBO bonding description above.

**Table 1:** Natural localised molecular orbital contributions to the principal ^13^C nuclear shielding components (*σ*^d^ + [*σ*^p^ + *σ*^so^]) of 1 and 2

Compound		SR			SOR			*Δ* ^so^ d			% NBO	Occ.
NLMO^a^		L^b^	NL^c^	L + NL	L^b^	NL^c^	L + NL	L^b^	NL^c^	L + NL		
1												
σ-Ce=C	***σ***_**iso**_	**−135**	**4**	**−131**	**−186**	**3**	**−183**	**−51**	**−1**	**−52**	**89**	**1.78**
	*σ*_11_	−432	20	−412	−495	18	−477	−63	−2	−65		
	*σ*_22_	3	−11	−8	−76	−13	−89	−79	−2	−81		
	*σ*_33_	25	3	28	13	3	16	−12	0	−12		
π-Ce=C	***σ***_**iso**_	**−123**	**15**	**−108**	**−121**	**16**	**−105**	**2**	**1**	**3**	**85**	**1.70**
	*σ*_11_	−405	35	−370	−400	32	−368	5	−3	2		
	*σ*_22_	−5	4	−1	2	9	11	7	5	12		
	*σ*_33_	42	8	50	35	7	42	−7	−1	−8		
2 × C–P	***σ***_**iso**_	**−57**	**−5**	**−62**	**−40**	**−5**	**−45**	**17**	**0**	**17**	**98**	**1.96**
	*σ*_11_	−9	2	−7	14	1	15	23	−1	22		
	*σ*_22_	−88	−12	−100	−60	−12	−72	28	0	28		
	*σ*_33_	−75	−5	−80	−73	−5	−78	2	0	2		
1s_core_-C^e^	***σ***_**iso**_	**200**	**0**	**200**	**204**	**0**	**204**	**4**	**0**	**4**	**100**	**2.00**
*Σ*_other_ f	***σ***_**iso**_	**−9**	**7**	**−2**	**−4**	**5**	**1**	**5**	**−2**	**3**	g	h

2^i^												
σ-Ce=C	***σ***_**iso**_	**−101**	**−11**	**−112**	**−227**	**−5**	**−232**	**−126**	**6**	**−120**	**89**	**1.79**
	*σ*_11_	−386	−4	−390	−563	4	−559	−177	8	−169		
	*σ*_22_	71	−30	41	−126	−20	−146	−197	10	−45		
	*σ*_33_	13	1	14	7	1	8	−6	0	−6		
π-Ce=C	***σ***_**iso**_	**−100**	**13**	**−87**	**−101**	**13**	**−88**	**−1**	**0**	**−1**	**85**	**1.71**
	*σ*_11_	−394	35	−359	−390	35	−355	4	0	4		
	*σ*_22_	13	0	13	7	0	7	−6	0	−6		
	*σ*_33_	82	5	87	81	5	86	−1	0	−1		
2 × C–P	***σ***_**iso**_	**−65**	**−10**	**−75**	**−24**	**−12**	**−36**	**41**	**−2**	**39**	**98**	**1.96**
	*σ*_11_	4	−2	2	54	−4	50	50	−2	48		
	*σ*_22_	−100	−23	−123	−27	−26	−53	73	−3	70		
	*σ*_33_	−99	−5	−104	−99	−5	−104	0	0	0		
1s_core_-C^e^	***σ***_**iso**_	**201**	**0**	**201**	**207**	**0**	**207**	**6**	**0**	**6**	**100**	**2.00**
*Σ*_other_ f	***σ***_**iso**_	**−11**	**10**	**−1**	**2**	**−1**	**1**	**13**	**−11**	**2**	g	h

a B3LYP calculations (HF = 20%, 1; 30% 2), all shielding parameters are in ppm.

b Lewis contribution of the NLMO.

c Non-Lewis contribution of the NLMO.

d Defined as *σ*(SOR) − *σ*(SR) to isolate the SO component.

e Essentially isotropic so only the average values provided.

f Minor component so only average values provided.

g Multiple NLMOs, but % NBOs all >85%.

h Multiple NLMOs, but all occupancies >1.71 electrons per NLMO.

i Data given for one carbene only since the data for both are identical.

Inspection of the data in Table 1 reveals that the principal shielding contributions to the *δ*_iso_ values of 1 and 2 are dominated by the Ce=C σ- and π-bond components supplemented by smaller C–P bond contributions. These contributions are in essence counter-balanced only by the *σ*^d^ contribution from the 1s core C_carbene_ orbital since various Lewis and Non-Lewis contributions from the Ce ions and other minor contributions tend to cancel out. Thus, like any other M=CR_2_ bond, the C_carbene_ centres in 1 and 2 exhibit *δ*_iso_ values that reflect stabilisation of the C_carbene_ by the metal- and R-substituents (where here R = the phosphonium groups).

Focussing on the SOR-NLMO-NMR aspects of Table 1, the data clearly show a dominance of the Ce=C bonds in total (1: −288 ppm; 2, −320 ppm) over the total two C–P bonds (1: −45 ppm; 2, −36 ppm) to the shielding. Thus, for 1 and 2 the M (here Ce) is performing the dominant stabilising role with the C–P constituting a much smaller stabilising role.

Where the split of σ- *vs.* π-bonding of the Ce=C bonds in 1 and 2 are concerned, in both cases the former component dominates over the latter, being ∼2 : 1 and ∼3 : 1, respectively. This confirms that the σ-components are strongest, but it is also the case that for 1 and 2 in each case the π-components are over twice that of the two C–P bonds *combined*. The Ce=C π-bonds are thus clearly far from being negligible, and weaker π-bonds compared to σ-bonds would anyway be anticipated from basic σ- and π-orbital overlap efficiency arguments. The presence of Ce=C π-bonds from this analysis is also consistent with the QTAIM data that consistently present non-zero ellipticity parameters consistent with the presence of double bonds rather than the zero ellipticity parameters that are associated with single and triple bonds.

Whilst the NLMO analysis does not report which virtual orbitals the NLMO orbitals are magnetically coupled to, the MO analysis provides the necessary framework to rationalise the NLMO shielding data. In particular, echoing the MO analysis the Ce=C σ- and π-bonds all exhibit large deshielded *σ*_11_ values, that are consistently the largest components of the breakdown of *σ*_iso_, resulting from σ/π* and π/σ* magnetic coupling that can be visualised as rotation of the relevant C p-orbitals about the *x* axis. Interestingly, for both complexes the C–P bonds show not insignificant deshielded *σ*_22_ and *σ*_33_ values, reflecting magnetic coupling that can be visualised as rotation of the C p-orbital aligned along the P–C–P bond (*x* axis) into the σ* (*z* axis, rotation about the *y* axis) and π* (*y* axis, rotation about the *z* axis), respectively. These contributions are facilitated by the T-shaped nature of the Ce=CP_2_ linkages in 1 and 2,^31^ but are still far smaller than the main Ce=C σ- and π-bond contributions to the shielding.

The NLMO analysis also reveals another interesting feature, which is that the σ-component is larger for 2 than for 1 even though in 2 there are two mutually *trans*-carbene donors; these strong σ-donors would ordinarily be anticipated to result in mutually weaker, not stronger, *trans* bonding. Thus, the shielding and bond order data presented here further support the presence of an ITI, which had previously been proposed for 2 on the basis of structural data and 5p-orbital in- and out-of-core calculations.^19^ This situation for 2 is accompanied by the π-bonding component still being present, but weaker than the π-bonding component in 1, which is in-line with the flexible nature of the bonding of these carbenes. This also likely reflects the dominance of the σ-bonding leaving the Ce ion with a diminished requirement for additional π-bonding compared to the situation in 1.

Since increased stabilisation is another way of articulating stronger bonding in covalent interactions, then given the relationship between shielding and *σ*^p^ and the NBO (and NLMO) bonding descriptions, these data support the presence of Ce=C double bonding interactions in 1 and 2.

The data in Table 1 also permits an analysis of the spin–orbit contributions by subtraction of the SR-NLMO values from the SOR-NLMO data. Notably, for 1 and 2 the dominant spin–orbit contributions are mediated by the Ce=C σ-bonds rather than the π-bonds. This can be rationalised by recalling that the 4f-orbitals will likely mediate the majority of the spin–orbit contributions from the Ce ion, and that transfer of spin–orbit-induced spin-polarisation to the NMR-nucleus (C_carbene_) will be *via* the C 2s orbital (Fermi contact). The NBO (and NLMO) data consistently show significant 4f contributions to the Ce-bonding, but variable 2s C-character. The Ce=C σ-bonds consistently show ∼11–15% C 2s character, which is evidently sufficient to mediate the spin-polarisation by Fermi contact, whereas the Ce=C π-bonds exhibit ≥98% 2p character and thus they have little 2s character to mediate spin–orbit contributions. Notably, the NLMO spin–orbit contribution for 2 is large, as it was for the *σ*^so^ value from the shielding analysis, for the Ce=C σ-bond. This further supports the presence of significant 4f-orbital character in the Ce=C bond, even though 4f-orbitals are normally regarded as being ‘core-like’, which is also consistent with the presence of ITI bonding in 2.

## Discussion

This study was prompted by the fact that diphosphonioalkylidenes have proven to be a highly effective ligand class for developing the M=C double bond chemistry of the lanthanides and early actinides, but the nature of these formal M=C interactions has been an open question due to the number of resonance forms that can be used to depict them. By undertaking computational analysis of experimental ^13^C NMR spectroscopic data of the two cerium(iv)–diphosphonioalkylidene complexes 1 and 2 the shielding values that underpin the experimentally observed *δ*_iso_ data have been computationally decomposed in detail, thus bringing quantitative benchmarks to a hitherto qualitatively descriptive framework.

Having identified DFT functionals that reproduce the ^13^C_carbene_*δ*_iso_ values to within 2 ppm, there can be confidence in the resulting computational benchmarking descriptions. It is interesting to note that whilst the BP86 functional does not reproduce the *δ*_iso_ values as well as B3LYP functionals, the MO, bond order, NBO, and NLMO analysis from either functional for 1 and 2 are, like-for-like, very similar. So whilst BP86 does not fare as well as B3LYP in the fine detail of reproducing shielding tensors, which are exceedingly sensitive to the computed wavefunction and spin–orbit effects, any differences recede with the arguably coarser orbital and bond order metrics. The tentative implication is that BP86 is adequate for ‘generic’ orbital and bond order analysis, but a hybrid functional really is needed for ‘specific’ sensitive spectroscopic parameters, a situation that was also found in modelling a terminal uranium(vi)-nitride.^58^

The consistent picture that emerges from the computational analysis is that the highly deshielded experimentally observed ^13^C_carbene_*δ*_iso_ values are predominantly due to large, negative *σ*^p^ values, which in themselves directly reflect strong Ce=C multiple bonds and external field-induced magnetic coupling of occupied and vacant orbitals associated with that linkage. Indeed, complexes 1 and 2 exhibit the largest ^13^C chemical shift tensor spans of any metal-alkylidene to date.^31–35^ The NLMO-NMR analysis reveals a dominance of Ce=C σ- over π-bond contributions, but these combined are far greater than the contributions from the phosphonium-substituents. The Ce=C π-bonds, perhaps the most debatable component of the bonding, are hence shown to be far more substantial than the two phosphonium-substituents combined, which together reaffirms the notion that the Ce=C bond is principally stabilised by the Ce ion, with the two phosphonium-substituents providing much weaker stabilisation. These data are all consistent with prior QTAIM data, whose ellipticity parameters were consistent only with the presence of Ce=C double bonds in 1 and 2. Indeed, the consistent picture that emerges from the MO and NLMO analysis is the dominance of the strongly deshielded *σ*_11_ component, which is a signature of alkylidenes.^31^

The NLMO analysis also clearly shows that the two Ce=C σ-bonds in 2 are evidently strong, which given they are mutually *trans* is notable and provides further support for the prior suggestion of the presence of an ITI in 2.^19^ The NLMO analysis also shows variable transmittance of spin–orbit-coupling, which can be related to the hybridisation of the alkylidene centres, *i.e.* greater 2s character facilitates greater *σ*^so^. That there are substantial spin–orbit contributions that likely originate from the Ce 4f-orbitals, which are usually described as ‘core-like’ and hence interacting little with ligand frontier orbitals, is notable and in-line with the overall description of 1 and 2 as exhibiting significant Ce=C double bonds. Lastly, the clearly larger spin–orbit contributions for 2 compared to 1 reflects the strong Ce=C σ-bonding, which again emphasises the presence of an ITI in this tetravalent complex.

## Conclusions

To conclude, assessing the shielding tensors that underpin the *δ*_iso_ values for 1 and 2 has enabled a detailed investigation of the nature of the Ce–BIPM interactions in these complexes, including confirming the Ce=C double bond character, revealing a record ^13^C chemical shift tensor span, signature deshielded *σ*_11_ components, and providing further support for the presence of an ITI in 2. The known ^13^C_carbene_ chemical shift range for f-element–diphosphonioalkyidene complexes spans over 300 ppm,^1–10^ and so clearly there is a wide variance of bonding situations, from highly ionic at low, shielded *δ*_iso_ values all the way to substantial covalent M=C double bonds at high, deshielded *δ*_iso_ values. This work confirms the M=C double bond credentials of f-element–diphosphonioalkylidene complexes, suggesting that mid-/high-valent uranium congeners also do indeed possess significant U=C double bonds,^38^ thus providing benchmarks towards the upper end of M=C double bonding character that will help provide an overall quantified framework for understanding diphosphonioalkylidene bonding generally.

## Methods

Magic angle spinning (MAS) NMR spectra were recorded on a Bruker 9.4 T (400 MHz ^1^H Larmor frequency) AVANCE III spectrometer equipped with a 4 mm HFX MAS probe that was used in ^1^H/^19^F/^13^C triple resonance mode. Experiments were acquired at ambient temperature using a MAS frequency of 8 or 11 kHz. ^1^H- and ^13^C-pulses of 100 kHz and 50 kHz were used, respectively, and spectra were recorded after {^1^H}^13^C cross-polarisation (CP, 4 ms for 1 and 2 ms for 2) and an echo sequence that used a free-evolution delay of 1 rotor period either side of the π-pulse. For CP, a 70–100% ramp was used for ^1^H to match 50 kHz ^13^C spin-locking. The ^1^H T_1_ was 0.14 s for 1 and 3.5 s for 2. Recycle delays of 1.0 and 4.6 s were used for 1 and 2, respectively and the {^1^H-}^13^C CPMAS NMR spectra were recorded for 88 and 65 hours, respectively. The ^13^C chemical shifts were referenced to TMS using an external reference sample. Spectral simulations were performed in the solid line-shape analysis (SOLA) module v2.2.4 in Bruker TopSpin v4.0.9. Samples were packed into 4 mm o.d. zirconia rotors in a glove box and sealed with a Kel-F rotor cap. Care must be taken with air sensitive compounds to minimise sample decomposition during measurements.

Restricted calculations were performed using the Amsterdam Density Functional (ADF) suite version 2017 with standard convergence criteria.^74,75^ Geometry optimisations were performed using coordinates derived from the respective crystal structures as the starting points. The H-atom positions were optimised, but the non-H-atom positions were constrained as a block. The DFT geometry optimisations employed Slater type orbital (STO) TZ2P polarisation all-electron basis sets (from the Dirac and ZORA/TZ2P database of the ADF suite). Scalar relativistic approaches (spin–orbit neglected) were used within the ZORA Hamiltonian^76–78^ for the inclusion of relativistic effects and the local density approximation (LDA) with the correlation potential due to Vosko *et al.* was used in all of the calculations.^79^ Generalised gradient approximation corrections were performed using the functionals of Becke and Perdew.^80,81^

Scalar and spin–orbit relativistic (ZORA-TZ2P-all-electron) single point energy calculations were then run on the geometry optimised coordinates. The conductor-like screening model (COSMO) was used to simulate benzene solvent effects. The functionals screened included BP86, PBE0-HF25, PBE0-HF40, B3LYP-HF20, B3LYP-HF30, B3LYP-HF35, and B3LYP-HF40, with B3LYP-HF20 and B3LYP-HF30 giving the closest agreement of computed NMR properties compared to experiment for 1 and 2, respectively. Nalewajski-Mrozek values were computed within the ADF program. NBO and NLMO analyses were carried out on the respective B3LYP data using NBO6.^82^ These calculations used the Hartree–Fock RI scheme to suspend the dependency key and overcome numerical issues. The MOs and NBOs were visualised using ADFView.

NMR shielding calculations were carried out using the NMR program within ADF.^72,73,83–87^ Calculated nuclear shieldings were converted to chemical shifts by subtraction from the calculated nuclear shielding of CH_4_ calculated at the same SR or SOR functional level in each case (HF20 SR/SOR = 191.3/192.1; HF30 SR/SOR = 191.4/192.2). MO contributions to the nuclear shieldings were calculated at the scalar and spin–orbit levels, the former with the FAKESO key. Scalar and spin–orbit NLMO calculations of the computed nuclear shieldings were carried out using NBO6 and ADF. These calculations used the Hartree–Fock RI scheme to suspend the dependency key and avoid numerical issues. Shielding tensors were visualised using TensorView.^88^

## Data availability

All data are available within this article, the ESI (Tables S1–S8†), or from S. T. L. on reasonable request.

## Author contributions

C. F. B. and J. A. S. prepared and purified the compounds and assisted in acquiring and analysing the solid-state NMR data. D. L. acquired and analysed the solid-state NMR data. R. W. A., D. L., and S. T. L. supervised and directed the research. S. T. L. conceived the central idea, conducted the DFT calculations, analysed the data, and wrote the manuscript with input from all the authors.

## Conflicts of interest

The author declares no competing interests.

## Supplementary Material

SC-015-D3SC04449A-s001
