# Supplementary material for: 13Ccarbene nuclear magnetic resonance chemical shift analysis confirms CeIV[double bond, length as m-dash]C double bonding in cerium(iv)–diphosphonioalkylidene complexes
Source: Chem Sci. 2023 Dec 6;15(1):238–49. doi: 10.1039/d3sc04449a (PMC10732143; doi:10.1039/d3sc04449a)
Supplement: SC-015-D3SC04449A-s001 [file SC-015-D3SC04449A-s001.pdf]

Electronic Supplementary Material (ESI) for Chemical Science.
This journal is © The Royal Society of Chemistry 2023

– Electronic Supporting Information –

$^{13}C_{carbene}$ Nuclear Magnetic Resonance Chemical Shift Analysis Confirms Ce^{IV}=C Double Bonding in Cerium(IV)-Diphosphonioalkylidene Complexes

Cameron F. Baker,[a] John A. Seed,[a] Ralph W. Adams,[a] Daniel Lee,*[,b] and Stephen T. Liddle*[,a]

[a] Department of Chemistry, The University of Manchester, Oxford Road, Manchester, M13 9PL, UK.

[b] Department of Chemical Engineering, The University of Manchester, Oxford Road, Manchester, M13 9PL, UK

*Email: daniel.lee@manchester.ac.uk; steve.liddle@manchester.ac.uk

Table S1. Geometry Optimised Coordinates (H-atoms optimised, non-H-atoms fixed) and Single Point Energy for 1.

1.H	-1.528358	-2.272888	-6.794111
2.H	-0.510760	-0.136036	-6.011719
3.C	-1.122946	-2.197505	-5.783637
4.C	-0.552835	-0.998569	-5.344916
5.H	-1.626236	-4.234661	-5.256357
6.H	-5.315166	-1.170325	-4.901383
7.C	-1.176745	-3.298822	-4.920794
8.H	-0.323043	2.318738	-4.899069
9.H	0.236701	4.004518	-4.859296
10.C	0.197694	3.067780	-4.283687
11.H	2.973566	-0.954001	-4.330569
12.C	-4.726867	-1.149849	-3.982292
13.H	-4.300452	0.966067	-4.113849
14.H	1.232020	2.732024	-4.129345
15.H	4.339585	0.163348	-4.117784
16.H	2.682831	0.795802	-4.209638
17.C	-0.032083	-0.902733	-4.051621
18.H	-4.973872	-3.262567	-3.586574
19.H	-2.517716	2.944264	-3.791238
20.C	3.295190	-0.032032	-3.824139
21.H	-2.045213	4.654733	-3.709399
22.H	0.420102	0.027145	-3.710164

23.C	-4.157553	0.048509	-3.541056
24.C	-0.659239	-3.203607	-3.626782
25.H	-0.010188	5.905769	-3.414161
26.C	-4.536238	-2.323627	-3.243566
27.H	2.006179	-3.723552	-3.294299
28.C	-1.991910	3.692241	-3.179251
29.H	3.454702	-5.679783	-2.849949
30.C	-0.070076	-2.004846	-3.184834
31.H	-0.704302	-4.067222	-2.961711
32.C	-0.530135	3.270336	-2.941827
33.C	0.384104	5.584906	-2.447318
34.H	-0.540509	2.305282	-2.417525
35.H	-2.532934	3.807723	-2.230548
36.C	2.158999	-4.056396	-2.267426
37.C	-3.406626	0.075036	-2.362773
38.H	1.183623	7.536943	-1.974547
39.C	2.980981	-5.158788	-2.016338
40.C	-3.783867	-2.299407	-2.066769
41.C	0.204759	4.256246	-2.042859
42.H	-2.968814	1.008530	-2.012640
43.H	4.417151	-2.395094	-1.957775
44.C	1.054762	6.506885	-1.642103
45.Si	3.206350	-0.179253	-1.939499
46.H	-3.642824	-3.218726	-1.496444
47.C	-3.221295	-1.093767	-1.609339
48.H	5.505817	-1.034074	-1.595830
49.H	4.722697	1.736431	-1.508323
50.H	3.036155	2.302512	-1.510490
51.C	4.500797	-1.446463	-1.407373
52.P	0.574089	-1.837190	-1.475415
53.N	1.579304	-0.534676	-1.349640
54.C	1.532913	-3.379425	-1.206557
55.C	3.697496	1.482698	-1.193963
56.C	3.199582	-5.586125	-0.702522
57.H	3.849704	-6.440537	-0.506647
58.C	0.722379	3.855825	-0.781438
59.H	-4.148193	2.771245	-0.796559
60.C	1.558314	6.097231	-0.406296
61.H	4.425849	-1.678696	-0.335310
62.H	-5.145568	1.394777	-0.276498
63.O	0.558395	2.575215	-0.370081
64.H	2.082649	6.819708	0.223032
65.H	3.702485	1.455668	-0.092797
66.C	-0.586168	-1.578810	-0.261246
67.C	1.407732	4.782044	0.050497
68.P	-2.188811	-1.039655	-0.093776
69.C	1.746450	-3.822757	0.108567
70.C	-4.526441	2.230009	0.082584
71.H	-5.086022	-1.668322	0.257649
72.C	2.581029	-4.915071	0.359540
73.H	-5.184072	2.918593	0.638138
74.Ce	0.364728	0.570339	0.399727

75.N	-2.033135	0.508171	0.456412
76.H	3.989159	3.952811	0.659187
77.H	1.251701	-3.303508	0.930372
78.H	-1.652915	3.657585	0.831644
79.C	-4.536100	-2.119855	1.083566
80.H	2.749833	-5.244605	1.385828
81.H	3.796421	5.579346	1.347098
82.C	-3.133954	-2.032150	1.127706
83.H	5.663665	0.313209	1.324749
84.Si	-3.134957	1.653335	1.228523
85.C	3.501246	4.525229	1.459886
86.C	1.970012	4.371548	1.404859
87.H	1.739476	3.306557	1.544753
88.H	5.284254	-2.186054	1.637532
89.H	3.361620	-0.602092	1.596667
90.H	-6.323940	-2.851268	2.043906
91.C	-2.100995	3.158181	1.703140
92.C	-5.235914	-2.785027	2.094357
93.H	4.630039	1.495614	2.167195
94.C	5.091478	0.501683	2.245100
95.H	-2.739209	3.892311	2.220789
96.H	3.888321	4.170368	2.426764
97.H	1.523443	6.221745	2.487951
98.C	-2.445872	-2.617809	2.202548
99.H	-1.289256	2.897997	2.400766
100.H	-1.357092	-2.559100	2.230581
101.C	4.033475	-0.595973	2.466462
102.C	4.704411	-1.979361	2.548921
103.C	1.307834	5.144953	2.560171
104.H	0.216491	5.020329	2.551695
105.O	1.250720	0.243674	2.361429
106.H	-4.715584	0.244673	2.605619
107.H	3.960160	-2.778088	2.669306
108.H	5.801198	0.530151	3.085725
109.C	-3.934716	0.992789	2.806133
110.C	-4.544083	-3.356195	3.167557
111.H	5.398624	-2.034888	3.400315
112.C	-3.147387	-3.270491	3.219741
113.H	1.691210	4.790893	3.528535
114.H	-4.401476	1.830716	3.349902
115.H	-3.194721	0.527084	3.472811
116.H	-5.091659	-3.867553	3.960966
117.C	1.817557	0.086370	3.586702
118.C	3.181701	-0.305700	3.693881
119.H	-0.744709	0.582777	3.648986
120.H	-2.603879	-3.714844	4.055055
121.H	-0.046272	2.844337	4.502808
122.C	-0.426703	0.690898	4.695462
123.C	1.045764	0.311374	4.761566
124.H	4.801591	-0.699205	5.057691
125.C	3.752211	-0.411248	4.969002
126.H	-1.706961	2.437647	5.005613

127.C	-0.648734	2.157070	5.112382
128.H	-1.181485	-1.295154	5.237683
129.H	-2.362446	0.018267	5.457086
130.C	-1.299276	-0.248263	5.548289
131.C	1.670587	0.189938	6.009320
132.H	-0.362409	2.308203	6.164177
133.C	3.015421	-0.159474	6.125730
134.H	-1.033226	-0.180950	6.613440
135.H	1.087116	0.373044	6.913729
136.H	3.482069	-0.242594	7.107387

Energy: –925.81129548 eV

Table S2. Geometry Optimised Coordinates (H-atoms optimised, non-H-atoms fixed) and Single Point Energy for 2.

1.C	2.456375	2.319414	-5.920675
2.C	-2.656080	-2.882090	-5.605792
3.C	2.970749	3.473823	-5.316885
4.C	2.072100	1.229237	-5.136742
5.C	-3.447135	3.155010	-5.346036
6.C	3.097157	-3.721217	-5.252866
7.C	-2.985443	-4.041036	-4.892621
8.C	-2.074080	3.095463	-5.093338
9.C	-2.304362	-1.714358	-4.924405
10.C	1.744174	-3.601089	-4.925741
11.C	4.007701	-2.753549	-4.808131
12.C	-4.300780	2.200615	-4.777245
13.C	-1.554569	2.092713	-4.266472
14.C	3.093853	3.532146	-3.924978
15.C	1.302140	-2.525243	-4.147564
16.C	3.562303	-1.675932	-4.041105
17.C	-3.779143	1.196873	-3.959732
18.C	2.194927	1.278744	-3.735968
19.C	2.205089	-1.556418	-3.691877
20.C	-2.957045	-4.025758	-3.493993
21.C	-2.400926	1.140448	-3.685031
22.C	-2.275310	-1.689306	-3.517816
23.C	2.705184	2.441671	-3.141745
24.C	-2.602973	-2.857502	-2.814043
25.C	-0.042651	-0.109323	-2.391406
26.C	5.225214	0.644908	-1.507800
27.C	-0.309326	3.820935	-1.507767
28.C	-5.170792	-1.136389	-1.216965
29.C	0.459655	-3.926552	-1.056363
30.C	4.325775	-2.109969	-0.498990
31.C	-4.522583	1.774944	-0.508947
32.C	2.122858	4.366491	0.281789
33.C	-1.960450	-4.332704	0.776535
34.C	-0.687881	5.321080	1.074794
35.C	3.893360	0.421122	1.183838
36.C	-3.803206	-0.497220	1.401485
37.C	0.863847	-5.129622	1.676166

38.C	0.058007	0.152089	2.384213
39.C	-2.585969	2.848874	2.721462
40.C	2.712004	-2.505832	3.055144
41.C	-3.741480	3.273180	3.382348
42.C	-1.443897	2.471957	3.442743
43.C	3.856584	-2.861997	3.773482
44.C	1.396872	2.513317	3.576080
45.C	1.572364	-2.023552	3.714731
46.C	1.459061	3.886097	3.876003
47.C	-1.259246	-2.054256	3.862545
48.C	-1.383128	-3.406629	4.227179
49.C	2.376002	1.661010	4.102365
50.C	-3.769005	3.324106	4.779986
51.C	-2.159606	-1.124840	4.398149
52.C	2.490855	4.395603	4.665997
53.C	-1.480833	2.523760	4.848053
54.C	-2.400397	-3.821225	5.088536
55.C	3.876379	-2.734805	5.166216
56.C	1.601537	-1.896969	5.115627
57.C	3.406936	2.168015	4.901254
58.C	-2.636108	2.945780	5.510911
59.C	3.469582	3.535645	5.181032
60.C	-3.172800	-1.535397	5.271711
61.C	-3.299074	-2.883996	5.614660
62.C	2.745898	-2.249459	5.835090
63.H	2.361869	2.264323	-7.006272
64.H	-2.680087	-2.883918	-6.696633
65.H	3.280078	4.321618	-5.930645
66.H	-3.853749	3.939170	-5.987381
67.H	3.443296	-4.561844	-5.857096
68.H	-3.268490	-4.950470	-5.425535
69.H	1.683156	0.329862	-5.617309
70.H	-1.400431	3.829086	-5.537804
71.H	-2.056558	-0.814712	-5.489998
72.H	5.064814	-2.835458	-5.065368
73.H	1.024764	-4.343404	-5.274135
74.H	-5.373137	2.235949	-4.975547
75.H	-0.483392	2.044487	-4.071222
76.H	0.245898	-2.432099	-3.897151
77.H	4.276632	-0.917972	-3.722195
78.H	3.496672	4.426046	-3.446739
79.H	-4.450482	0.448844	-3.539652
80.H	-3.216471	-4.922988	-2.930339
81.H	5.281688	0.415104	-2.580904
82.H	-5.263512	-1.024803	-2.306805
83.H	2.801505	2.482896	-2.058239
84.H	-0.110226	4.772862	-2.025400
85.H	0.196348	3.026392	-2.074594
86.H	-2.584538	-2.843388	-1.725838
87.H	5.111638	1.734885	-1.413811
88.H	-0.053291	-3.202029	-1.704571
89.H	4.430572	-2.545586	-1.503674

90.H	-1.391221	3.630197	-1.563385
91.H	0.271735	-4.929552	-1.471394
92.H	6.198007	0.376919	-1.062795
93.H	-4.692765	2.106452	-1.543321
94.H	-4.954058	-2.196267	-1.017315
95.H	-6.157154	-0.914422	-0.776745
96.H	1.539143	-3.730410	-1.135107
97.H	2.289763	5.297644	-0.283591
98.H	5.281508	-2.246770	0.032400
99.H	2.728730	3.575380	-0.183219
100.H	-3.812149	2.479823	-0.054450
101.H	-2.102480	-5.309852	0.287067
102.H	3.558189	-2.694222	0.028473
103.H	-5.476453	1.858805	0.037089
104.H	-2.589604	-3.597640	0.254244
105.H	-0.426626	6.258168	0.555252
106.H	2.508074	4.514556	1.301621
107.H	-1.772415	5.175085	0.963492
108.H	3.707872	1.505297	1.189086
109.H	0.591607	-6.126137	1.289840
110.H	-2.342811	-4.410358	1.804912
111.H	-3.479930	-1.540825	1.528797
112.H	4.875172	0.247236	1.652056
113.H	-2.557018	2.811971	1.633759
114.H	1.944189	-5.000147	1.514874
115.H	3.135135	-0.045790	1.828168
116.H	-4.796436	-0.395999	1.867381
117.H	-0.486483	5.474336	2.144105
118.H	-3.107955	0.136658	1.970673
119.H	2.691941	-2.608321	1.971781
120.H	-4.618257	3.568608	2.804582
121.H	0.698140	-5.142412	2.762509
122.H	4.730832	-3.243870	3.244515
123.H	0.691178	4.561352	3.500860
124.H	-0.677949	-4.142644	3.843945
125.H	2.324102	0.593473	3.890749
126.H	-2.064487	-0.073301	4.128906
127.H	2.527656	5.463466	4.886530
128.H	-4.668041	3.661502	5.298574
129.H	-2.488260	-4.875476	5.355513
130.H	-0.600767	2.238504	5.426702
131.H	4.766376	-3.019604	5.730113
132.H	4.158133	1.488050	5.305044
133.H	0.722152	-1.530530	5.647704
134.H	-3.863137	-0.796140	5.679997
135.H	4.273934	3.932246	5.803365
136.H	-4.092234	-3.206435	6.291927
137.H	-2.648760	2.986763	6.601183
138.H	2.751532	-2.152773	6.921819
139.Ce	0.018106	0.003922	-0.002861
140.N	2.240047	-0.031237	-1.181808
141.N	-2.261171	-0.169975	-1.067988

142.N	0.013946	2.323818	0.984166
143.N	0.101055	-2.175075	1.258122
144.P	1.610026	-0.125978	-2.692550
145.P	-1.706726	-0.185379	-2.610810
146.P	0.033165	1.824968	2.545473
147.P	0.094924	-1.487784	2.746244
148.Si	3.860627	-0.274388	-0.561273
149.Si	-3.878996	-0.005155	-0.408106
150.Si	0.285878	3.903840	0.271717
151.Si	-0.134517	-3.826568	0.722720

Energy: −1102.38595417 eV

Table S3. Computed Scalar Relativistic (SR) and Spin-Orbit Relativistic (SOR) $^{13}C_{carbene}$ Isotropic Chemical Shift (δ_{iso}), Isotropic Shielding (σ_{iso}), Diamagnetic Shielding (σ^d), Paramagnetic Shielding (σ^p), and Spin-Orbit Shielding (σ^{so}) Values for 1 Computed With Various Functionals.

Functional	*δ_{iso} Expt.*	*δ_{iso} Calc'd*	*σ_{iso}*	*σ^d*	*σ^p*	*σ^{so}*
BP86-SR	324.6	277	−88.8	272.5	−361.3	-
BP86-SOR		294.6	−105.6	272.5	−361.4	−16.7
B3LYPHF20-SR		298.4	−111.4	268.6	−380.0	-
B3LYPHF20-SOR		***324.9***	***−137.1***	***268.6***	***−382.1***	***−23.6***
B3LYPHF30-SR		276.3	−89.2	268.5	−357.7	-
B3LYPHF30-SOR		302.8	−114.9	268.5	−359.2	−24.2
B3LYPHF35-SR		257.5	−70.3	268.5	−338.8	-
B3LYPHF35-SOR		283.0	−95.1	268.5	−339.6	−24
B3LYPHF40-SR		236.7	−49.4	268.5	−317.9	-
B3LYPHF40-SOR		260.5	−72.5	268.5	−317.9	−23.1
PBE0HF25-SR		264.1	−72.4	270.0	−342.4	-
PBE0HF25-SOR		286.7	−94.2	270.0	−343.6	−20.6
PBE0HF40-SR		218.6	−21.3	269.1	−290.4	-
PBE0HF40-SOR		237.3	−39.3	269.1	−289.9	−18.5

Table S4. Computed Scalar Relativistic (SR) and Spin-Orbit Relativistic (SOR) $^{13}C_{carbene}$ Isotropic Chemical Shift (δ_{iso}), Isotropic Shielding (σ_{iso}), Diamagnetic Shielding (σ^d), Paramagnetic Shielding (σ^p), and Spin-Orbit Shielding (σ^{so}) Values for 2 Computed With Various Functionals. Note, data are an average of the two carbenes, where the variance between each carbene pair of values was ≤0.4 ppm.

Functional	*δ_{iso} Expt.*	*δ_{iso} Calc'd*	*σ_{iso}*	*σ^d*	*σ^p*	*σ^{so}*
BP86-SR	343.5	247.8	−59.6	277.4	−337.0	-
BP86-SOR		265.3	−76.3	277.4	−335.9	−17.8
B3LYPHF20-SR		271.7	−84.7	258.6	−343.3	-
B3LYPHF20-SOR		319.6	−131.8	258.6	−344.0	−46.4
B3LYPHF30-SR		258.9	−71.8	258.9	−330.7	-
B3LYPHF30-SOR		***341.8***	***−153.9***	***258.9***	***−333.1***	***−79.7***
B3LYPHF35-SR		249.1	−61.9	259.2	−321.1	-
B3LYPHF35-SOR		356.5	−168.6	259.2	−324.4	−103.4
B3LYPHF40-SR		237.8	−50.5	259.4	−309.9	-
B3LYPHF40-SOR		371.3	−183.3	259.4	−314.0	−128.7

PBE0HF25-SR	250.6	−58.9	258.3	−317.2	-
PBE0HF25-SOR	303.8	−111.3	258.3	−318.1	−51.5
PBE0HF40-SR	222.4	−29.4	258.6	−288.0	-
PBE0HF40-SOR	311.3	−117.6	258.6	−289.8	−86.4

Table S5. Comparison of NBO Data for the Ce=C Bond in 1 Using BP86 and B3LYP-HF20 Functionals.

Bond	*Functional*	*Ce%*	*C%*	*Ce 6s/6p/5d/4f*	*C 2s/2p*
Ce=C σ	BP86	13	87	3/0/21/76	12/88
	B3LYPHF20	13	87	1/0/32/67	15/85
Ce=C π	BP86	12	88	0/1/19/80	2/98
	B3LYPHF20	11	89	1/1/31/67	2/98

Table S6. Comparison of NBO Data for the Ce=C Bond in 2 Using BP86 and B3LYP-HF30 Functionals.

Bond	*Functional*	*Ce%*	*C%*	*Ce 6s/6p/5d/4f*	*C 2s/2p*
Ce=C σ	BP86	13	87	1/0/46/53	12/88
	B3LYPHF30	15	85	3/0/47/50	11/89
Ce=C π	BP86	8	92	0/1/19/80	0/100
	B3LYPHF30	7	93	0/0/53/47	0/100

Table S7. Comparison of NBO and NLMO Data for the Ce=C Bond in 1 Using the B3LYP-HF20 Functional.

Bond	*Representation*	*Ce%*	*C%*	*Ce 6s/6p/5d/4f*	*C 2s/2p*
Ce=C σ	NBO	13	87	1/0/32/67	15/85
	NLMO	12	77	0/0/31/69	15/85
Ce=C π	NBO	11	89	1/1/31/67	2/98
	NLMO	9	76	1/1/32/66	3/97

Table S8. Comparison of NBO and NLMO Data for the Ce=C Bond in 2 Using the B3LYP-HF30 Functional.

Bond	*Representation*	*Ce%*	*C%*	*Ce 6s/6p/5d/4f*	*C 2s/2p*
Ce=C σ	NBO	15	85	3/0/47/50	11/89
	NLMO	14	76	2/0/21/77	12/88
Ce=C π	NBO	7	93	0/0/53/47	0/100
	NLMO	6	80	0/1/53/46	0/100
